# Noninvasive ventilation and high-flow oxygen therapy for severe community-acquired pneumonia

**DOI:** 10.1097/QCO.0000000000000715

**Published:** 2021-01-18

**Authors:** Salvatore Lucio Cutuli, Domenico Luca Grieco, Luca Salvatore Menga, Gennaro De Pascale, Massimo Antonelli

**Affiliations:** a Dipartimento di Scienza dell’Emergenza, Anestesiologiche e della Rianimazione, Fondazione Policlinico Universitario Agostino Gemelli IRCCS, Largo Agostino Gemelli 8; b Facoltà di Medicina e Chirurgia ‘Agostino Gemelli’, Università Cattolica del Sacro Cuore, Largo Francesco Vito 1, Rome, Italy

**Keywords:** acute respiratory failure, high-flow nasal cannula oxygen therapy, hypercapnia, hypoxemia, noninvasive mechanical ventilation

## Abstract

We review the evidence on the use of noninvasive respiratory supports (noninvasive ventilation and high-flow nasal cannula oxygen therapy) in patients with acute respiratory failure because of severe community-acquired pneumonia.

**Recent findings:**

Noninvasive ventilation is strongly advised for the treatment of hypercapnic respiratory failure and recent evidence justifies its use in patients with hypoxemic respiratory failure when delivered by helmet. Indeed, such interface allows alveolar recruitment by providing high level of positive end-expiratory pressure, which improves hypoxemia. On the other hand, high-flow nasal cannula oxygen therapy is effective in patients with hypoxemic respiratory failure and some articles support its use in patients with hypercapnia. However, early identification of noninvasive respiratory supports treatment failure is crucial to prevent delayed orotracheal intubation and protective invasive mechanical ventilation.

**Summary:**

Noninvasive ventilation is the first-line therapy in patients with acute hypercapnic respiratory failure because of pneumonia. Although an increasing amount of evidence investigated the application of noninvasive respiratory support to hypoxemic respiratory failure, the optimal ventilatory strategy in this setting is uncertain. Noninvasive mechanical ventilation delivered by helmet and high-flow nasal cannula oxygen therapy appear as promising tools but their role needs to be confirmed by future research.

## INTRODUCTION

Despite recent improvements in the management of acute respiratory failure (ARF) and sepsis associated with lung infections, severe community-acquired pneumonia (SCAP) remains an issue for ICU physicians and a threat for affected critically ill patients [1^▪▪^,^2^]. Specifically, pneumonia represents the main cause for ICU admission among patients with acute respiratory failure, especially when underlying chronic obstructive pulmonary dysfunction (COPD) is present [3].

The mainstay of SCAP management includes appropriate antimicrobial therapy and respiratory supports delivered by either invasive or noninvasive means, when clinically relevant lung dysfunction occurs [4]. Moreover, ongoing coronavirus disease-2019 pandemic has underlined the outstanding relevance of noninvasive approaches in both respiratory support and microbiological diagnosis [5^▪^,^6^,7^▪^].

Accordingly, we reviewed the latest evidence on the use of noninvasive respiratory supports (noninvasive ventilation and high-flow nasal cannula oxygen therapy), aiming to define the role of such therapy in the integrated multidisciplinary approach to critically ill patients with respiratory failure because of SCAP. 

**Box 1:**
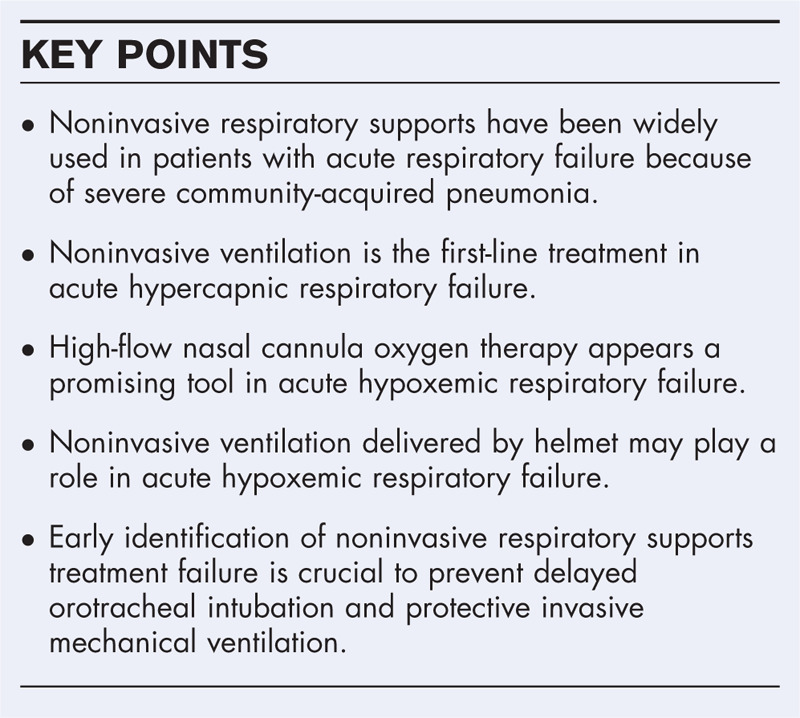
no caption available

## SEVERE COMMUNITY-ACQUIRED PNEUMONIA: DEFINITION, DIAGNOSIS AND TREATMENT LANDMARKS

The first challenge in the management of CAP is the early assessment of severity degree, as prompt recognition of respiratory failure, sepsis and septic shock is of paramount importance to prevent further deterioration and progression to multiorgan failure [8,9]. Pneumonia Severity Index, CURB-65 (confusion, blood urea nitrogen, respiratory rate, blood pressure and age >65 years) and quick Sequential Organ Failure Assessment (SOFA) have been demonstrated to efficiently identify patients requiring hospitalization [10]. For hospitalized patients, American Thoracic/Infectious Disease Societies (ATS/IDSA) major and minor criteria, including tachypnea, arterial O_2_ partial pressure (PaO_2_)/inspired oxygen fraction (FiO_2_) ratio and hypotension, are accurate clinical tools for ICU admission [1^▪▪^]. Early adequate antimicrobial therapy, including antibiotics active against typical/atypical agents and antivirals in epidemic settings are associated with improved outcome and should be based on international guidelines and local epidemiological features [11]. Along with the core pathogens involved in CAP, the new concept of PES (*Pseudomonas aeruginosa*, extended-spectrum beta-lactamase producing *Enterobacteriales* and methicillin-resistant *S. aureus*) pathogens has been recently developed [12^▪^]: accordingly, the identification of risk factors for such bacteria, including prior antibiotics, recent hospitalization, recent *P. aeruginosa*/MRSA infection or colonization, poor functional status and immune suppression, should drive a broader spectrum antibiotic approach [13]. Nonetheless, steroids in SCAP, when viral etiology is excluded and inflammation markers are elevated, should be promptly used for blunting inflammation-driven organ failure [14]. In this sense, an accurate microbiological diagnosis is strongly advocated in critically ill patients and the choice of invasive (bronchoalveolar lavage and endotracheal aspirate) vs. noninvasive (sputum, urinary antigens, serology, nasal and throat swabs, blood cultures) diagnostic tools may influence the type of respiratory assistance (invasive vs. noninvasive ventilation) and oxygenation (high or low-flow) therapy [15^▪^]. Additionally, the use of standard microbiology techniques may lack of accuracy, especially when performed on sputum and upper airway samples [16]. Moreover, recent antibiotic administration may limit culture-based pathogen identification. Accordingly, culture-independent/point-of-care diagnostic tools should be implemented in clinical practice with the purpose of overcoming such issues. [6,17].

From a clinical perspective, ARF in patients with SCAP is characterized by acute onset (within a week) symptoms and gas exchange impairment (hypoxemia and hypercapnia). Dyspnea and tachypnea (defined as respiratory rate >25 breaths/min and/or active contraction of accessory respiratory muscles) are present in a wide proportion of patients.

Gas exchange impairment allows the identification of two different pathophysiological pathways that are hypercapnia or hypoxemia (although both conditions may coexist).

Acute hypercapnic respiratory failure is most commonly observed in patients with COPD and is complicated by an exacerbation of the underlying disease. It is diagnosed arterial pH less than 7.35 and CO_2_ partial pressure (PaCO_2_) above 45 mmHg. Dyspnea may not always be present, especially when PaCO_2_ is increased enough to induce changes in mental states.

Acute hypoxemic respiratory failure is most commonly observed in de novo ARF and is diagnosed by radiological evidence of pulmonary infiltrates of noncardiogenic origin, hypoxemia with normocapnia or hypocapnia [18]. Hypoxemia is defined by PaO_2_/FiO_2_ (ratio of arterial oxygen partial pressure to the fraction of inspired oxygen) of 300 mmHg or lower, which can be replaced by the ratio of SpO_2_/FiO_2_ (ratio of peripheral oxygen saturation to the fraction of inspired oxygen) of 315 mmHg or lower [19]. This corresponds to PaO_2_ less than 60 mmHg or SpO_2_ less than 90% while the patient breaths ambient air.

During ARF, increased intrapulmonary shunt is the main mechanism for hypoxemia, whereas hypoventilation and increased alveolar dead space are the main mechanisms for hypercapnia [20].

**FIGURE 1:**
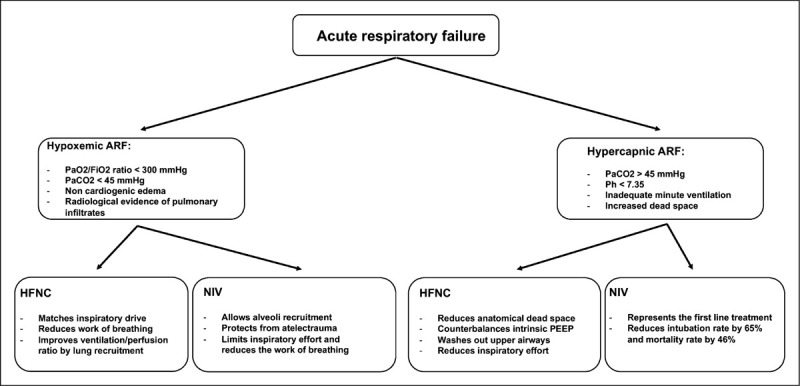
Diagnostic and therapeutic strategies of acute respiratory failure.

The differentiation of these two entities has relevant clinical implications, as the choice, setting and efficacy of the noninvasive respiratory supports critically depends on the underlying pathophysiological process [21] (Fig. 1).

## NONINVASIVE RESPIRATORY SUPPORTS

Noninvasive respiratory supports include therapeutic strategies that allow effective CO_2_ clearance and O_2_ supplementation through devices applied to the external surface of the upper airways (nose, mouth or both). Such tools have gained importance in the last decades because of negligible invasiveness (which allows prevention of complications associated with tracheal intubation like laryngeal trauma and tracheal impairment), improvement of patient comfort (e.g. nutrition, interaction with the environment) and preservation of physiological protective pathways (e.g. cough and clearance of secretions) [21]. Noninvasive respiratory supports are classified as high-flow nasal cannula oxygen (HFNCO) therapy and noninvasive ventilation (NIV).

## HIGH-FLOW NASAL CANNULA OXYGEN THERAPY

### Interfaces and settings

HFNCO therapy allows spontaneous breathing and delivers a high flow (up to 100 l/min, conventionally 30--60 l/min) of fully actively humidified air/oxygen (FiO_2_ ranging from 0.21 to 1.0) mixture via a single limb connected to nasal cannulas [22]. To prevent ambient contamination with aerosol, a surgical face mask can be applied to the face of the patient without altering HFNCO mechanism of action [23].

### Mechanism of action

The provision of heat and humified air reduces metabolic expenditure of the organism [24], prevents damage of the nasopharyngeal mucosa, preserves mucociliary function and secretions clearance. High gas flow matches patient's inspiratory flow [25^▪^], enabling accurate delivery of set FiO_2_. This yields important diagnostic (reliable assessment of PaO_2_/FiO_2_ ratio and lung dysfunction) and therapeutic advantages (improvement of oxygenation and consequent dyspnea relief).

HFNCO therapy improves upper airway conductance through the generation of continuous positive pressure [26] and provides flow-dependent, inter-individually variable, positive end-expiratory pressure (PEEP) in the lower airways [27], which is capable of improving ventilation to perfusion ratio by alveolar recruitment.

**FIGURE 2:**
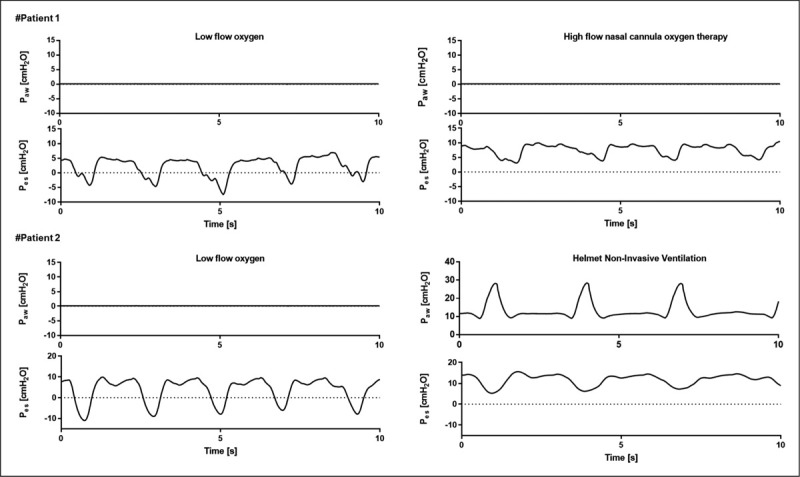
Sample tracings of airway pressure (*P*_aw_) and esophageal pressure (*P*_es_) of patients affected by acute hypoxemic respiratory failure. Patient no. 1 is severely tachypneic and shows high inspiratory effort in low-flow oxygen therapy (Δ*P*_es_ = 10--12 cmH_2_O). After the initiation of treatment with HFNCO, there is a reduction in the inspiratory effort and in the respiratory rate (Δ*P*_es_ 5--7 cmH_2_O). Patient no. 2 presents higher inspiratory effort then patient no. 1 (Δ*P*_es_ 15--20 cmH_2_O), consequently Helmet NIV was initiated by the treating physician, providing a marked reduction in Δ*P*_es_ (5--10 cmH_2_O). HFNCO, high-flow nasal cannula oxygen.

Finally, HFNCO therapy reduces anatomical dead space by washing CO_2_ out from upper airway [28], with reduction of dyspnea, inspiratory effort and minute ventilation [29] (Fig. 2).

### Main settings

Three settings are needed for HFNCO therapy: flow, FiO_2_ and temperature. The PEEP effect is proportional to the delivered flows; hence in patients for whom alveolar recruitment is the goal of HFNCO therapy, it is advisable to use the maximum tolerated flow. The benefit on CO_2_ washout is achieved with 30–45 l/min and is poorly enhanced by further increases in flow: in hypercapnic patients, this flow setting could represent the optimal choice [30]. Gas temperature does not change the clinical effect of HFNCO therapy, and should be individualized based on patient's comfort [31]. FiO_2_ should be set to maintain PaO_2_ between 55 and 70 mmHg or SpO_2_ between 88 and 92% [32].

### Noninvasive ventilation

NIV allows the delivery of positive pressure ventilation without bypassing the upper airways. In the ICU, NIV is delivered through oronasal masks, full-face masks and the helmet interface. Helmet internal volume is around 18 l and a gas flow ranging between 30 and 50 l/min is needed to prevent rebreathing [33]. Helmet NIV has other important features compared with other interfaces. First, minute ventilation monitoring is not reliable as part of such volume distends the interface and does not have any active contribution to patient's ventilation. Second, system pressurization is slowed by intrinsic elastic characteristics of the interface and the delay is proportional to the compliance of the system, which may potentially impair inspiratory muscles unloading [34] and improve lung recruitment [35^▪▪^].

Regardless of the interface used, the main assumption to deliver effective NIV relies on the close fitting between interface and body surfaces, in order to provide targeted positive inspiratory pressure and PEEP.

### Mechanism of action

NIV has important respiratory and hemodynamic implications, which arise from the delivery of energy into upper and lower airways that modify intrathoracic distribution of pressure gradients [21].

PEEP ameliorates the conductance of upper airways and increases oxygenation by improving ventilation to perfusion ratio through alveolar recruitment. Furthermore, it exerts protective effects on atelectrauma by reducing alveolar opening and closing with the respiratory cycle. Moreover, PEEP increases transpulmonary pressure, reduces venous return and lowers transmural ventricular pressure, thus reducing pulmonary edema and improving cardiac function in patients with congestive heart failure.

Inspiratory pressure unloads respiratory muscles, which reduces work of breathing, and together with PEEP, limits inspiratory efforts, thus modulating the risk of patient self-inflicted lung injury [36^▪^] (Fig. 2). Finally, PEEP improves dynamic hyperinflation and positive inspiratory pressure favors physiologic minute ventilation, thus improving CO_2_ washout [37].

### Main settings

During NIV, spontaneous breathing is assisted in both inspiratory and expiratory phases of the ventilatory cycle. NIV may be delivered by setting the mechanical ventilator in pressure support or bilevel modality. Such ventilatory approach allows the setting of different parameters:

(1) FiO_2_: it may range between 0.21 and 1.
(2) PEEP: it should be set according to patient's need and may be helped by esophageal pressure monitoring [38] and impedance tomography examination [39]. Although high PEEP may not be set in oronasal/facemask NIV because of a linear relationship with air leaks, in helmet NIV such relationship is inverse by improving adherence to patient's shoulders [40].
(3) Positive inspiratory pressure: this parameter should be tailored to patient inspiratory effort in order to provide optimal ventilatory assistance, thus reducing the risk of excessive inspiratory effort and over-assistance, with associated lung overdistention and diaphragmatic dysfunction. Patient inspiratory effort is best measured by esophageal pressure monitoring [38]. During helmet NIV, high positive inspiratory pressure allows interface distension and improves gas washout.
(4) Pressurization rate: this parameter should be set according to an inverse relationship with patient's inspiratory drive. Accordingly, it should be as short as tolerated by the patient, with the aim of matching patient's inspiratory flow, thus allowing respiratory muscles unloading [41].
(5) Gas conditioning: this feature may be controlled by either active heated humidifiers or heat and moisture exchangers. The latter has the inconvenience of adding dead space to the circuit, thus increasing the risk of hypercapnia. Both tools have the aim to reach an absolute humidity of 15 mgH_2_O/l [42]. However, recent evidence suggests that gas conditioning is not required during helmet NIV [43].
(6) NIV has some clinical contraindications, that include impaired consciousness, vomiting/gastrointestinal bleeding, recent facial or gastroesophageal surgery [21]. Moreover, NIV is associated with patient's discomfort because of skin damage and claustrophobia as well as life-threatening complications associated with its failure.

## ACUTE HYPERCAPNIC RESPIRATORY FAILURE

Acute hypercapnic respiratory failure is a life-threatening clinical condition, which frequently occurs in patients with COPD exacerbation and pneumonia [44,45]. Its severity correlates with mortality and is conditioned by both arterial CO_2_ level and acidemia [46,47]. CO_2_ is an acid and its blood accumulation lowers pH [37], especially when renal function does not cope efficiently in increasing the alkaline reserve of the organism. CO_2_ accumulation arises from an imbalance between metabolic production and respiratory elimination. From an epidemiological point of view, the latter is the most frequent condition, and is caused by insufficient minute ventilation (e.g. respiratory muscle dysfunction – pump failure – and low tidal volume) or increased dead space [37].

Hypercapnia and acidosis may induce arterial hypotension by reducing vascular sensitivity to catecholamines, altered organ perfusion, pulmonary arterial hypertension and inflammatory dysregulation [48]. Nonetheless, CO_2_ may be stored in extravascular tissues like muscles, fat and bones. These compartments are characterized by different equilibration times, thus causing delayed correction of CO_2_ imbalance, which may require even more than 48 h [49]. Accordingly, hypercapnia and acidosis represent a clinical emergency, and management relies on the efficacy of ventilatory strategies to revert underlying pathophysiological pathways.

### Noninvasive ventilation

During NIV, external PEEP reduces the additional pressure load because of intrinsic PEEP that must be surmounted to generate inspiratory flow, whereas positive inspiratory pressure helps to overcome the increased respiratory resistance related to COPD exacerbation. As external PEEP can further limit expiration and positive inspiratory pressure increases tidal volume and worsens hyperinflation, great caution is required when setting the ventilator. Minimal PEEP (3–5 cmH_2_O) is sufficient in most cases, whereas the level of positive inspiratory pressure should be titrated to achieve tidal volumes in the range of 6–8 ml/kg of predicted body weight (PBW); moreover, setting the cycling off criteria at a high percentage of peak inspiratory flow, if not generating double triggering, may contribute to prolonged exhalation, and mitigate hyperinflation. It must be noted that air leaks are frequent during NIV and cause delay in expiratory trigger activation, possibly limiting exhalation time and contributing to air trapping. The best-fitting mask can minimize air leaks and newer ventilators provide dedicated NIV modes allowing the setting of a time-regulated safety cycling off criteria.

NIV is the first-line treatment for acute hypercapnic respiratory failure because of COPD exacerbations, obesity, neuromuscular disease and chest wall deformity [44,45]. A recent systematic review and meta-analysis on patients with acute hypercapnic respiratory failure demonstrated that NIV compared with usual care decreases the risk of mortality by 46% and reduces the risk of tracheal intubation by 65% [50]. Moreover, NIV reduced the PaCO_2_ levels, the length of hospital stay, the incidence of complications not associated with such therapy, and improved pH as well as PaO_2_ levels [50]. Specifically, in patients with acute hypercapnic respiratory failure and SCAP [51], NIV was associated with significant reduction of endotracheal intubation and length of ICU stay. Moreover, in a subgroup of patients with COPD, NIV was associated with 2-months improved survival. Among more than 900 patients enrolled in RCTs, positive inspiratory pressure-NIV reduced the need for tracheal intubation by 65% and led to an overall reduction of 55% of in-hospital mortality, with increasing benefit with lower pH at inclusion [52]: such evidence is corroborated by observational data indicating similar efficacy outside the procedures of RCTs [53]. The number of patients needed to treat in order to avoid tracheal intubation and death are, respectively, around 4 and 10, making NIV in hypercapnic patients one of the most efficient treatments in ICU. Until other data emerge, a NIV trial through oronasal or face mask should be considered as the gold standard for the first-line treatment of acute hypercapnic respiratory failure.

### High-flow nasal cannula oxygen therapy

HFNCO therapy is a novel tool and its effect on reducing anatomical dead space and counterbalancing intrinsic PEEP has been linked with improved CO_2_ washout. In the light of this view, some studies indicated a potential role of HFNCO therapy in patients with acute hypercapnic respiratory failure. Rittayamai *et al.*[54] observed a reduction of inspiratory effort associated with a gas flow of 30 l/min, which was comparable with NIV delivered in positive inspiratory pressure modality (median positive inspiratory pressure 11 cmH_2_O and PEEP 5 cmH_2_O) in a cohort of 12 patients with COPD exacerbation. Furthermore, Sklar *et al.*[55] observed that diaphragm-thickening fraction measured by ultrasound was comparable between HFNCO therapy and NIV in a cohort of 15 patients with cystic fibrosis who developed acute hypercapnia. HFNCO therapy provided effective CO_2_ washout and respiratory rate decrease when compared with low-flow oxygen therapy in 48 patients with stable COPD [56]. Moreover, HFNCO therapy was more comfortable, although less effective, than NIV in reducing systemic CO_2_ levels in 24 patients with similar characteristics [57].

HFNCO therapy represents a promising alternative to NIV in the management of acute hypercapnic respiratory failure; however, its effect on clinical outcomes has never been assessed and warrants further investigations.

## ACUTE HYPOXEMIC RESPIRATORY FAILURE

In patients with hypoxemic respiratory failure Acute Respiratory D, NIV generates a significant oxygenation improvement thanks to the recruiting effect of applied PEEP [58]. Whenever tested as an alternative to direct intubation in patients failing oxygen therapy, positive inspiratory pressure-NIV allowed similar oxygenation improvement and provided the advantage of averting tracheal intubation in two-thirds of the treated patients, with an overall decrease in the occurrence of infectious complications during the ICU stay [58].

Nevertheless, other investigations did not confirm this benefit [59,60] and NIV failure rate ranges between 30 and 50%, with a higher likelihood in patients with most severe oxygenation impairment [60–65]. Importantly, patients failing NIV (and HFNCO therapy) are burdened by increased mortality [61,63,66,67], likely as a consequence of the prolonged exposure of injured lungs to spontaneous breathing and increased respiratory drive [60,68–71].

A strong physiological evidence supports HFNCO therapy as the optimal strategy to administer oxygen to hypoxemic critically ill patients with high respiratory demand. Accordingly, HFNCO therapy has been suggested as an alternative to NIV in patients with acute hypoxemic respiratory failure: in a RCT, HFNCO therapy was not inferior to positive inspiratory pressure-NIV sessions in preventing tracheal intubation but a post hoc analysis revealed a possible beneficial effect by HFNCO therapy in the subgroup of patients with PaO_2_/FiO_2_ less than 200 mmHg [73].

Different interfaces are available for NIV: face masks have been used in all the RCTs discussed so far, but helmets have the potential advantage of improving patients’ comfort, allowing the application of high PEEP with minimal air leaks during prolonged treatments without interruptions [72–74]. High PEEP lessens lung strain and atelectrauma, may reduce the driving pressure and convincingly improves mortality in intubated patients with early ARDS [74–77]: recent data indicate that high PEEP generates lung recruitment, mitigates lung injury and exerts similar beneficial physiological effects also during spontaneous breathing [78,79]. Delivery of high PEEP is difficult during face-mask NIV while it appears feasible when helmets are used, with the possibility of exploiting all the benefits of the noninvasive approach. In this sense, helmet continuous positive airway pressure (CPAP) with PEEP = 10 cmH_2_O, as compared with low-flow oxygen therapy was able to prevent the need for tracheal intubation in a small pilot randomized controlled trial involving hypoxemic patients with pneumonia [80]. Most importantly, a recent randomized controlled trial comparing helmet and full-face mask for positive inspiratory pressure-NIV and CPAP in patients with ARDS showed a huge benefit in the intubation rate (20 vs. 60%) and 90-day mortality in patients who received Helmet-NIV who, accordingly, underwent treatment with higher PEEP values [81]. Recently, a physiologic study showed that helmet NIV may improve oxygenation and mitigate inspiratory effort also when compared with HFNCO [82]. These results appear more hypothesis-generating than definite, given the small population and the monocentric design; however, the signal concerning the benefit of administering high PEEP in continuous sessions with improved comfort appears consistent with physiology and promising, warranting further investigations.

All these considerations explain why recent guidelines have been unable to provide recommendations on the use of NIV in patients with hypoxemic respiratory failure [44].

It appears wise to suggest that NIV and HFNCO therapy are well tolerated and effective in patients with PaO_2_/FiO_2_ greater than 200 mmHg, whereas patients with PaO_2_/FiO_2_ less than 200 mmHg represent the most at-risk population and should be treated with caution, in order to limit the risks of delayed intubation.

Consequently, monitoring is essential for the early detection of treatment failure: the ROX index and its trend over time, which assesses the ratio of SpO_2_/FiO_2_ to respiratory rate, may help early detect treatment failure [83,84].

In patients with hypoxemic respiratory failure, NIV may expose patients to barotrauma and volutrauma, both favored by the disproportionate increase of respiratory drive and asynchronization between patient and mechanical ventilator. During face-mask NIV, the likelihood of treatment failure may be predicted by increased tidal volume (>9.5 ml/kg of predicted body weight [85]) and by a composite scale including heart rate, acidosis, consciousness, oxygenation and respiratory rate (HACOR) [86,87].

## CONCLUSION

The choice, timing and setting of the noninvasive respiratory supports should be carefully tailored to patients’ specific requirements. Among patients with hypercapnic respiratory failure, NIV through oronasal and facemask greatly reduces the need for tracheal intubation and convincingly improves survival: the role of high-flow nasal cannula oxygen therapy remains to be established.

During acute hypoxemic respiratory failure, the optimal initial approach is hotly debated: whereas avoidance of tracheal intubation convincingly improves survival, maintenance of spontaneous breathing may worsen lung injury and mortality, especially in patients with a PaO_2_/FiO_2_ less than 200 mmHg, who seems to be the most delicate population. Recently, HFNCO therapy and high-PEEP Helmet NIV have been proposed to optimize the treatment and may foster NIV success but the best balance between these two techniques remains to be established and the patients that best benefit from the different approaches have yet to be identified. Careful clinical judgement is hence required during any treatment and physiological parameters may help to distinguish early, the patients with a high likelihood of success from those prone to failure, in whom prompt intubation and protective ventilation are mandatory.

## Acknowledgements

*None.*

### Financial support and sponsorship

*None.*

### Conflicts of interest

*There are no conflicts of interest.*

## REFERENCES AND RECOMMENDED READING

Papers of particular interest, published within the annual period of review, have been highlighted as:

▪ of special interest
▪▪ of outstanding interest
